# The Role of Vimentin in Human Corneal Fibroblast Spreading and Myofibroblast Transformation

**DOI:** 10.3390/cells13131094

**Published:** 2024-06-25

**Authors:** Miguel Miron-Mendoza, Kara Poole, Sophie DiCesare, Emi Nakahara, Meet Paresh Bhatt, John D. Hulleman, Walter Matthew Petroll

**Affiliations:** 1 Department of Ophthalmology, UT Southwestern Medical Center, Dallas, TX 75390, USA; 2 Department of Biomedical Engineering, UT Southwestern Medical Center, Dallas, TX 75390, USA

**Keywords:** cornea, vimentin, cytoskeleton, fibroblasts, myofibroblasts, wound healing, TGFβ, PDGF

## Abstract

Vimentin has been reported to play diverse roles in cell processes such as spreading, migration, cell–matrix adhesion, and fibrotic transformation. Here, we assess how vimentin impacts cell spreading, morphology, and myofibroblast transformation of human corneal fibroblasts. Overall, although knockout (KO) of vimentin did not dramatically impact corneal fibroblast spreading and mechanical activity (traction force), cell elongation in response to PDGF was reduced in vimentin KO cells as compared to controls. Blocking vimentin polymerization using Withaferin had even more pronounced effects on cell spreading and also inhibited cell-induced matrix contraction. Furthermore, although absence of vimentin did not completely block TGFβ-induced myofibroblast transformation, the degree of transformation and amount of αSMA protein expression was reduced. Proteomics showed that vimentin KO cells cultured in TGFβ had a similar pattern of protein expression as controls. One exception included periostin, an ECM protein associated with wound healing and fibrosis in other cell types, which was highly expressed only in Vim KO cells. We also demonstrate for the first time that LRRC15, a protein previously associated with myofibroblast transformation of cancer-associated fibroblasts, is also expressed by corneal myofibroblasts. Interestingly, proteins associated with LRRC15 in other cell types, such as collagen, fibronectin, β1 integrin and α11 integrin, were also upregulated. Overall, our data show that vimentin impacts both corneal fibroblast spreading and myofibroblast transformation. We also identified novel proteins that may regulate corneal myofibroblast transformation in the presence and/or absence of vimentin.

## 1. Introduction

The cytoskeleton is composed of three filamentous proteins: F-actin, microtubules, and intermediate filaments (IFs). These cytoskeletal filament proteins form highly structured and dynamic networks. Cytoskeletal filament proteins reorganize quickly in response to external and internal signals, allowing cells to adapt their shape in response to environmental or chemical cues [1,2]. These changes in cell shape are important in cell biological processes such as cell division, migration, adhesion, and the response to external forces [3,4,5,6,7]. The three cytoskeletal filament proteins differ not only in their chemical and physical structure but also in the types of filaments and networks they form [1]. While the role of F-actin and microtubules in the spreading and morphology of cells is well established, questions remain regarding the functional roles of IFs. The family of proteins forming IFs is vast, they are differentially expressed, and the form and structure of the network often depend on IF type, cell type, as well as differentiation status [8].

Vimentin is a type III IF protein expressed mainly in mesenchymal cell types [9]. Fibroblasts are cells of mesenchymal origin that are broadly found in many tissues and organs [10]. Vimentin intermediate filaments, VIFs, are generally distributed homogenously throughout the cytoplasm of fibroblasts [11]. Recent studies have shown that VIFs in fibroblasts play diverse roles in cell mechanical processes [12], such as modulating cell extensions [13], regulating migration and movement [14], contributing to cell growth and development of cell–matrix adhesions [15], and maintaining cellular integrity [9]. VIFS have also been shown to play a role in cancer invasiveness [16,17], wound repair [18,19,20,21], and fibroblast aging [22]. VIFs also provide the cells with mechanical support to stabilize them and allow them to withstand large amounts of stress without damage [8,23]; VIFs are elastic polymers that can withstand deformations of up to 300% of their initial length [23]. The precise mechanisms of action through which VIFs function have not been fully elucidated and can vary depending on cell type and culture conditions.

The cornea is an optically transparent tissue at the front of the eye that is responsible for two-thirds of its refractive power. The corneal stroma, which makes up 90% of the corneal thickness, is a highly ordered structure consisting of lamellae of aligned collagen fibrils that endow the tissue with its mechanical stability and optical transparency. Corneal keratocytes are sandwiched between these collagen lamellae and interact with the extracellular matrix (ECM) to regulate fundamental biological processes such as developmental morphogenesis and wound healing. Dysregulated wound healing can result in corneal fibrosis and scarring, which is a leading cause of blindness worldwide [24,25]. Stromal haze is caused by activation of keratocytes into migratory fibroblasts which repopulate the wound and transform into myofibroblasts, which secrete a disorganized fibrotic ECM [25,26].

Recent studies have shown that a decrease in the steady-state levels of vimentin or inhibition of vimentin phosphorylation using the pharmacological agent Withaferin inhibit myofibroblast differentiation of corneal keratocytes, resulting in decreased fibrosis following corneal injury in a mouse model [20,21,27]. In this study, we explored the role of VIFs in modulating both spreading and myofibroblast transformation of cultured human corneal fibroblasts. First, we developed vimentin knockout (Vim KO) and knockdown (Vim KD) cells and analyzed their spreading and morphology on 2D substrates and in 3D collagen matrices. We also analyzed the effect of Withaferin on PDGF-induced cell spreading and tractional force generation. Subsequently, we analyzed the effect of vimentin depletion on myofibroblast transformation of corneal fibroblasts and conducted proteomics analysis to compare the patterns of protein expression between vimentin KO cells and control cells.

## 2. Materials and Methods

### 2.1. Materials

Dulbecco’s modified Eagle medium (DMEM) and transforming growth factor beta (TGFβ) were obtained from Sigma-Aldrich (St. Louis, MO, USA). OptiMEM was obtained from Gibco (Grand Island, NY, USA). Fibroblast growth media and fibroblast growth kit-low serum were obtained from ATCC (Gaithersburg, MD, USA). The 0.25% trypsin/EDTA solution and RNAiMAX transfection reagent were purchased from Invitrogen (Gaithersburg, MD, USA). Platelet-derived growth factor BB isotype (PDGF) was obtained from Upstate Biotechnology, Inc. (Lake Placid, NY, USA). Fetal bovine serum (FBS), fatty acid-free, and fraction V bovine serum albumin (BSA), penicillin, streptomycin, and amphotericin B were obtained from Lonza (Walkersville, MD, USA). Withaferin A was obtained from Abcam (Waltham, MA). Vimentin siRNA was purchased from ThermoFisher (Waltham, MA, USA). Non-targeting siRNA and siTOX transfection control were obtained from Horizon Discovery Biosciences (Cambridge, UK). Cas9/tracRNA/crRNA RNP and vimentin primers were obtained from Integrated DNA Technologies (Coralville, IA, USA). Pierce RIPA buffer and the Protease and Phosphatase Inhibitor cocktail were obtained from Thermo Scientific (Waltham, MA, USA). Mini-protean TGX gels, PVDF membrane, and HRP-conjugated goat anti-mouse antibody were obtained from Bio-Rad (Hercules, CA, USA). Type I rat tail collagen high concentration was purchased from BD Biosciences (Bedford, MA, USA). Alexa Fluor 488, 546, 633, and propidium iodide (PI) were obtained from Molecular Probes, Inc. (Eugene, OR, USA). RNase (DNase-free) was purchased from Roche (Indianapolis, IN, USA). Vimentin antibody, vimentin-conjugated 488 and 546, and GAPDH antibody were obtained from Santacruz (Dallas, TX, USA). FITC goat-anti-rabbit-conjugated and goat-anti-mouse antibody were obtained from Jackson ImmunoResearch (West Grove, PA, USA).

### 2.2. Cell Culture

We used an established cell line (HTK) in which the catalytic subunit of human telomerase (h-TERT) was used to extend the lifespan of corneal fibroblasts [28]. HTK cells were maintained in tissue culture flasks at 37 °C in a 5% CO_2_ humidified incubator using DMEM containing 10% FBS and supplemented with 1% penicillin, 1% streptomycin, and 1% amphotericin B.

All experiments were carried out with serum-free basal media (SF), composed of DMEM and supplemented with essential amino acids, ascorbic acid, vitamins, and 5 mg/mL of BSA. PDGF-BB (50 ng/mL) or TGFβ1 (5 ng/mL) was added to media to study the cell response to growth factors. For cell spreading experiments, media containing PDGF was used and incubations were carried out for 4 and 24 h. In some experiments, Withaferin was also added at a concentration of 2 μM. SF media was used as control. For myofibroblast transformation, cells were incubated for 6 days with media containing TGFβ, and SF media was used as control. Collection of protein for Western blotting was carried out after the 6th day of incubation with TGFβ1.

### 2.3. Vimentin Knockdown

For siRNA vimentin reverse transfection, a vimentin siRNA master mix solution was prepared by combining transfection reagent RNAiMAX at a ratio of 1:100 with OptiMEM. Vimentin siRNA was added at a final concentration of 100 nM. The vimentin siRNA solution was poured into a well plate. An equal amount of solution containing trypsinized cells (200,000 cells) was poured on top of the same well and mixed with siRNA solution. For control experiments, vimentin siRNA was replaced with non-targeting siRNA or siTOX. After 24 h of incubation, the media was changed to DMEM. Following 72 h of incubation, a second reverse transfection was carried out in the same way as described above. The media was changed to DMEM after 24 h of incubation, and after 72 h, cells were collected for experiments.

### 2.4. Vimentin Knockout

For vimentin knockout experiments, HTK cells were electroporated with Cas9/tracRNA/crRNA RNP (1350 V, 30 ms, single pulse). The crRNA used was Hs.Cas9.Vim.1.AB (Seq: GGACTCGGTGGACTTCTCGC, PAM: TGG). Electroporated cells were screened using VIM exon 2 primers to confirm editing. Heterogeneous cell populations were sorted by flow cytometry into 96-well plates. Single cells were grown in fibroblast basal medium supplemented with the low-serum growth kit. The fastest-growing clones were scaled up and analyzed for Vim KO via qPCR, EnGen, and Western blotting. Clones were then expanded and cultured for experiments.

### 2.5. 3D Collagen Matrix Model

A neutralized collagen solution was prepared by mixing high-concentration rat tail type I collagen with 0.1 N of NaOH, 10XMEM, H_2_O, and DMEM containing cells at a density of 5 × 10^4^ cells/mL to achieve a final collagen concentration of 2 mg/mL. Next, 150 µL of solution was poured on Mattek glass dishes. Samples were then placed into a humidified incubator (37 °C, 5% CO_2_) for 60 min for polymerization. After samples polymerized, media containing PDGF were added to stimulate cell spreading.

### 2.6. Collagen Matrix Contraction

Collagen matrices were prepared as described above. Immediately after samples polymerized and media were added, matrix height was measured. Samples were returned into the humidified incubator and incubated for 24 h. Matrix height was measured again. Height is measured by focusing on the top and bottom of each matrix. Since the bottom of the matrix remains attached to the dish, the change in height is a measure of the matrix reorganization produced by the cells, expressed as percentage of matrix contraction. Duplicate samples were analyzed for each condition in each experiment.

### 2.7. Immunostaining

Samples were fixed using 3% paraformaldehyde in phosphate-buffered saline (PBS) for 10 min and permeabilized with 0.5% Triton X-100 in PBS for 15 min. Samples were then washed with PBS and blocked with 1% BSA fraction V in PBS for 1 h. For vimentin labeling, samples were incubated with a vimentin monoclonal antibody for 120 min, washed for 60 min, and then incubated with an FITC-conjugated goat anti-mouse secondary antibody. Samples were washed for 60 min and then incubated with Alexa-fluor 546 Phalloidin (1:150 ratio) to label F-actin. After 60 min of washing, samples were incubated for 30 min with DAPI (1:100 ratio) in PBS containing 1:100 RNase-DNase free to label cell nuclei. All staining processes were carried out on the same plates used for the experiments to avoid cell or matrix distortion.

Fluorescence images were collected with a laser confocal microscope (Leica SP8, Heidelberg, Germany). An Argon laser (488 nm) was used to visualize vimentin, a GreNe laser (543 nm) was used for F-actin, and a UV laser (405 nm) was used to image nuclei. Images were acquired sequentially to avoid cross talk between the channels. A stack of optical sections was acquired by changing the position of the focal plane in the z-direction with a step size of 1 or 2 µm, using a 63× oil immersion objective or 25× water immersion objective, respectively.

### 2.8. Immunoblotting

Protein was extracted with a lysis buffer containing Pierce RIPA buffer and the Protease and Phosphatase Inhibitor cocktail at a 100:1 ratio. Lysates were clarified at 10,000× *g* at 4 °C for 10 min. Protein was subjected to SDS-PAGE electrophoresis using a Bio-Rad electrophoresis chamber and mini-protean TGX gels. Transfer to PVDF membranes was carried out with a trans-blot turbo transfer system from Bio-Rad. Blots were probed with mouse anti-alpha smooth muscle actin, anti-GAPDH, and anti-vimentin antibodies followed by HRP-conjugated goat anti-mouse antibodies. ImageQuant TL software (version 8.1) was used to quantify protein expression, which was normalized to GAPDH.

### 2.9. Image Processing and Analysis

Imaging processing of collected images was carried out using MetaMorph software version 7.7 (Molecular Devices, Inc., San Jose, CA, USA). Z-stack images from each channel were integrated into single maximum-intensity projection images and combined using the color overlay function. For morphometric measurements, Image J software (version 1.54d) was used to outline cells (manually) and calculate cell area, length, and aspect ratio.

### 2.10. Statistical Analysis

All statistical analysis was performed using GraphPad Prism 10. Two-way ANOVA was used to compare group means, and post hoc multiple comparisons tests were performed using the Sidak or Tukey methods. A *p*-value of less than 0.05 was considered statistically significant.

### 2.11. Proteomics

Protein was extracted as for Western blotting, as described previously. Protein was subjected to SDS-PAGE electrophoresis in mini-protean TGX gels for 20 min to cluster protein. Gels were stained with Coomassie dye for 1 h and then washed twice with de-stain solution for 30 min. Gel bands were diced into 1 mm³ cubes and placed in Eppendorf tubes. Samples were digested overnight with trypsin (Pierce) following reduction and alkylation with DTT and iodoacetamide (Sigma–Aldrich). The samples then underwent solid-phase extraction cleanup with an Oasis HLB plate (Waters) and the resulting samples were injected onto a QExactive HF mass spectrometer coupled to an Ultimate 3000 RSLC-Nano liquid chromatography system. Samples were injected onto a 75 um i.d., 15 cm long EasySpray column (Thermo, Waltham, MA, USA) and eluted with a gradient from 0 to 28% buffer B over 90 min with a flow rate of 250 nL/min. Buffer A contained 2% (*v/v*) ACN and 0.1% formic acid in water, and buffer B contained 80% (*v/v*) ACN, 10% (*v/v*) trifluoroethanol, and 0.1% formic acid in water. The mass spectrometer operated in positive ion mode with a source voltage of 2.5 kV and an ion transfer tube temperature of 275 °C. MS scans were acquired at 120,000 resolution in the Orbitrap and up to 20 MS/MS spectra were obtained for each full spectrum acquired using higher-energy collisional dissociation (HCD) for ions with charges 2–8. Dynamic exclusion was set for 20 s after an ion was selected for fragmentation.

Raw MS data files were analyzed using Proteome Discoverer v.3.0 (Thermo), with peptide identification performed using Sequest HT searching against the human-reviewed protein database from UniProt. Fragment and precursor tolerances of 10 ppm and 0.02 Da were specified, and three missed cleavages were allowed. Carbamidomethylation of Cys was set as a fixed modification, and oxidation of Met was set as a variable modification. The false discovery rate (FDR) cutoff was 1% for all peptides.

## 3. Results

### 3.1. Knockout of Vimentin Alters PDGF-Induced Elongation of Corneal Fibroblasts

#### 3.1.1. Corneal Fibroblast Spreading on 2D Substrates

**Figure 1:**
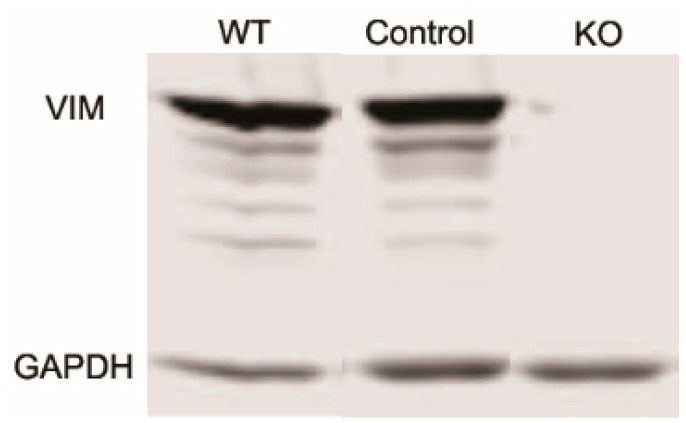
Western blot showing a lack of vimentin protein expression in vimentin knockout (KO) cells as compared to wild-type (WT) and controls.

Studies have shown that vimentin intermediate filaments (VIFs) can modulate cellular extensions and contribute to cell movement and migration in other cell types [13,14]. To study the role of VIFs in the spreading of corneal fibroblasts, we proceeded to develop vimentin knockout (Vim KO) cells, culture them, and evaluate their spreading. We verified that vimentin is not present in Vim KO cells using PCR and Western blotting (Figure 1).

**Figure 2:**
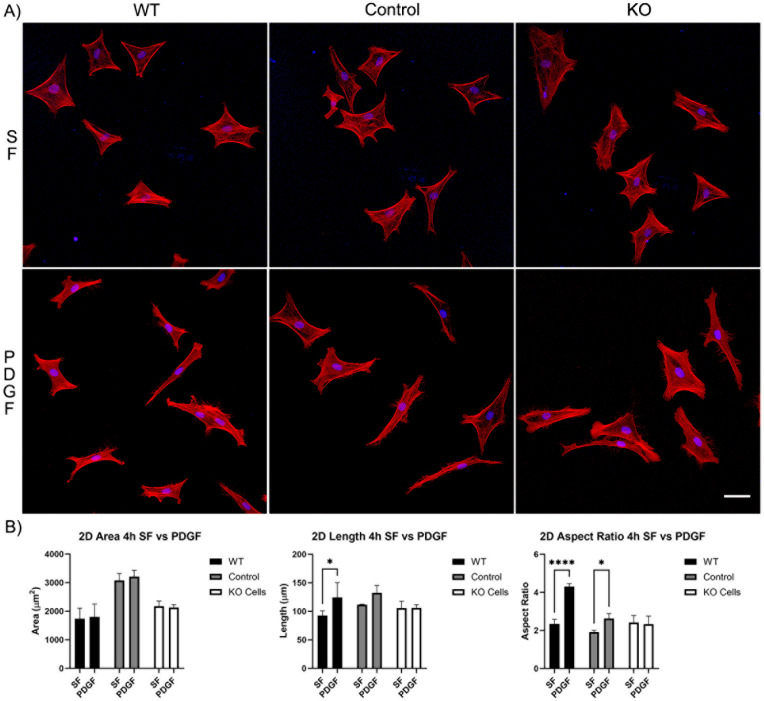
The effect of Vim KO on corneal fibroblasts spreading on 2D substrates following culture for 4 h in the presence of serum-free (SF) media with or without the addition of PDGF. (**A**) Representative pictures of samples that were fixed and labeled for F-actin (red) and nuclei (blue). Scale bar is 50 μm. (**B**) Graphs showing morphological data from 3 independent experiments. Error bars show standard deviations. (* *p* < 0.05, **** *p* < 0.0001).

Our first set of experiments was carried out on 2D substrates with media containing PDGF or serum-free (SF) basal media. Cells were incubated for 4 and 24 h. After just 4 h of incubation, wild-type cells, editing control cells, and Vim KO cells were all able to spread normally in SF media (Figure 2A). Although, the cell area for the editing control cells was significantly higher than the cell area of wild-type and Vim KO cells (Figure 2B). The addition of PDGF-BB induced an increase in cell length and/or the degree of elongation (aspect ratio) in wild-type and control cells, consistent with previous studies. However, Vim KO cells did not show significant elongation in response to PDGF.

**Figure 3:**
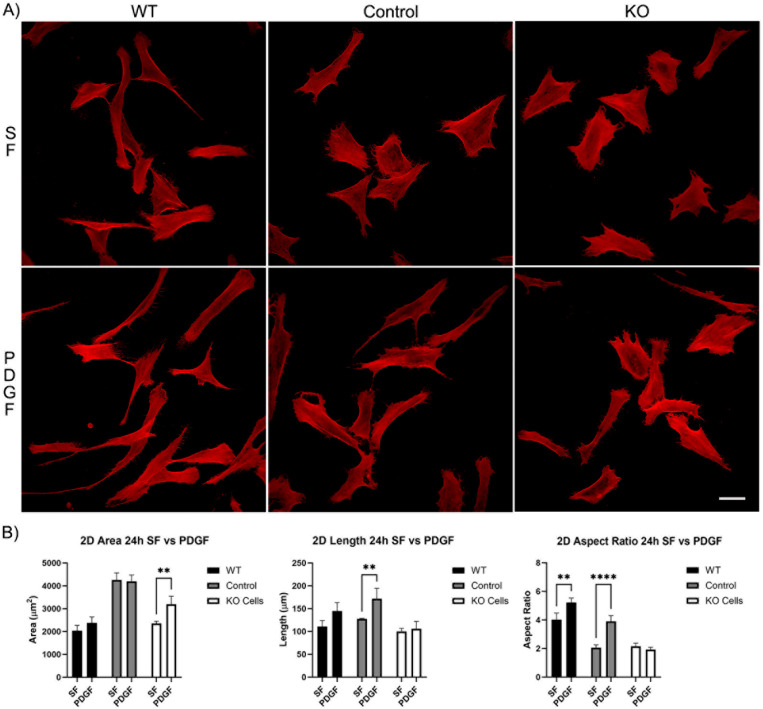
The effect of Vim KO on corneal fibroblasts spreading on 2D substrates following culture for 24 h in the presence of serum-free (SF) media with or without the addition of PDGF. (**A**) Representative pictures of samples that were fixed and labeled for F-actin (red). Scale bar is 50 μm. (**B**) Graphs showing morphological data from 3 independent experiments. Error bars show standard deviations. (** *p* < 0.01, **** *p* < 0.0001).

Longer periods of incubation (24 h) showed that all cells continued to spread and elongate (Figure 3A). However, the difference in response of the Vim KO cells to PDGF was even more pronounced, as no increase in either the length or aspect ratio was observed (Figure 3B).

#### 3.1.2. Corneal Fibroblast Spreading in 3D Collagen Matrices

**Figure 4:**
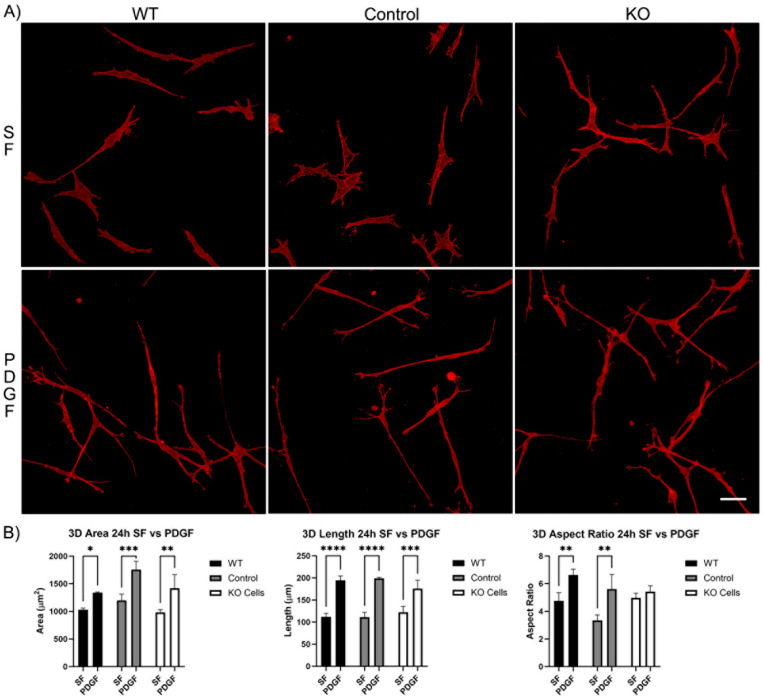
Vim KO cell spreading in 3D collagen matrices for 24 h in the presence of PDGF and SF media. (**A**) Representative pictures of samples that were fixed and labeled for F-actin. Scale bar is 50 μm. (**B**) Graphs are morphological data from 3 different experiments. (* *p* < 0.05, ** *p* < 0.01, *** *p* < 0.001, **** *p* < 0.0001).

We next evaluated the spreading of Vim KO cells in 3D collagen matrices. After 4 h of incubation, wild-type cells, editing control cells, and Vim KO cells were all able to spread within 3D matrices (Figure S1A). In SF media, the cell area and length were similar for all three cell types. However, PDGF-induced elongation was higher in wild-type cells as compared to both editing control and Vim KO cells (Figure S1B). Incubation in 3D collagen matrices for 24 h showed that all cell types continued to spread and elongate (Figure 4A). The addition of PDGF induced an increase in cell length and the degree of elongation (aspect ratio) in wild-type and control cells. However, Vim KO cells did not show significant elongation in response to PDGF (Figure 4B).

Previous studies in our lab have shown that cell polarization and elongation is a common response induced by PDGF [29]. The results of both the 2D and 3D cell spreading experiments in the current study suggest that while the ability of cells to spread is generally maintained in the absence of vimentin, the elongation response is somewhat mitigated in Vim KO cells.

### 3.2. Vim KO and Wild-Type Cells Produce Similar Amounts of Matrix Reorganization

**Figure 5:**
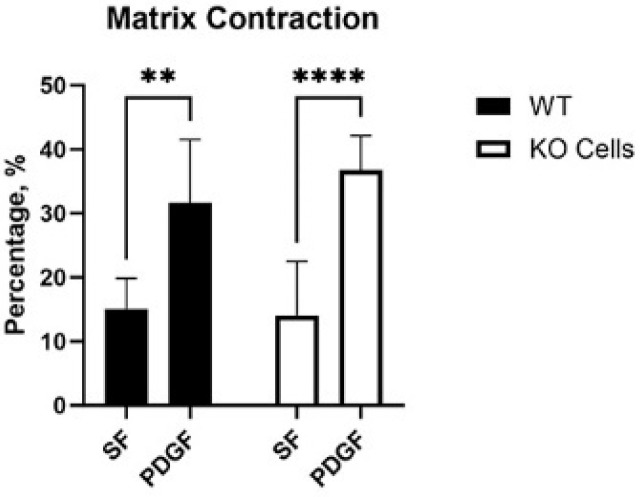
Collagen matrix contraction induced by wild-type (WT) and Vim KO cells. Graphs are data from 3 different experiments. (** *p* < 0.01, **** *p* < 0.0001).

Cells exert tractional forces on the matrix during spreading. To test if Vim KO cells exert similar amounts of tractional force as wild-type cells, we used a collagen matrix contraction assay. In this assay, the height of an attached collagen matrix is reduced over time due to cell-induced matrix reorganization. Interestingly, wild-type and KO cells induced similar amounts of matrix contraction in SF media, and a significant increase was observed in response to PDGF in both cell types (Figure 5).

### 3.3. Knockdown of Vimentin Does Not Alter Corneal Fibroblast Spreading

**Figure 6:**
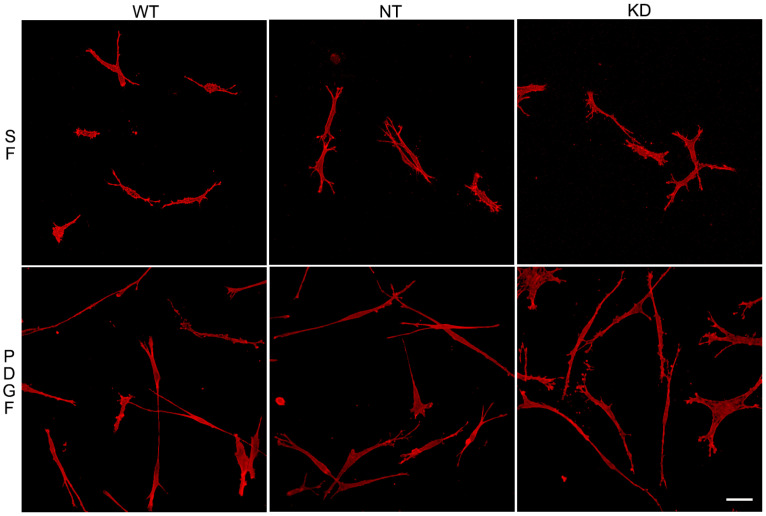
Vim KD cell spreading in 3D collagen matrices for 24 h in the presence of PDGF and SF media.Representative pictures of cells that were fixed and labeled for F-actin. Scale bar is 50 μm.

To assess whether the ability of Vim KO cells to continue to spread in the absence of the protein was due to compensatory mechanisms developed over time, we evaluated the effect of transient vimentin knockdown on corneal fibroblast spreading. Knockdown of 82% was achieved (Figure S2). Vim KD cells, non-targeting control cells (NT), and wild-type cells were incubated in 3D collagen matrices as described above and labeled for F-actin. Interestingly, no obvious changes in the spreading of Vim KD cells and their response to PDGF were observed (Figure 6), consistent with our results showing only subtle changes in cell spreading in Vim KO cells.

### 3.4. Blocking Vimentin Polymerization Impacts Cell Spreading and Tractional Force Generation

**Figure 7:**
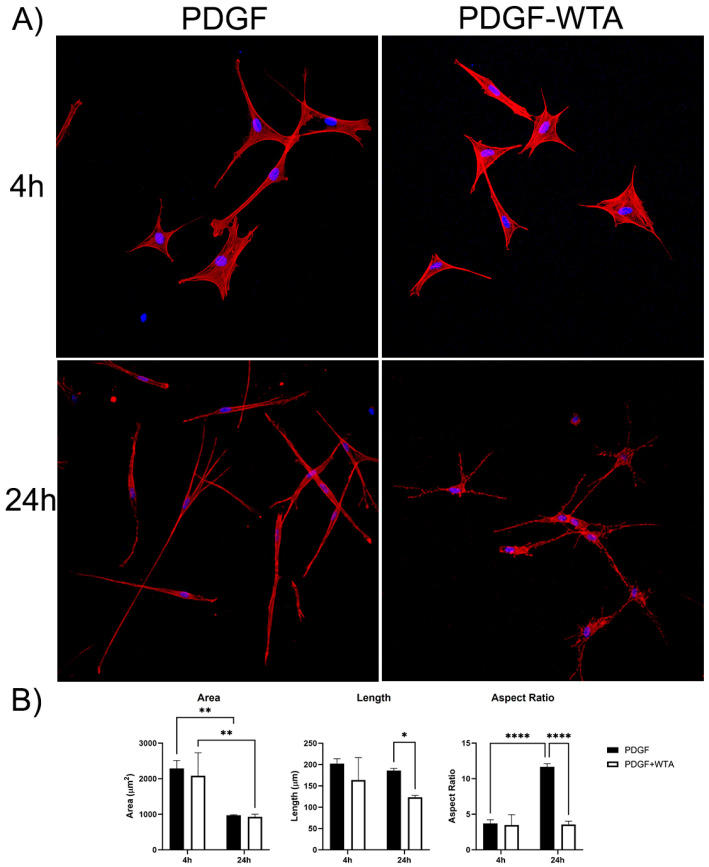
(**A**) Representative pictures of initial cell spreading of corneal fibroblasts cultured on 2D collagen-coated dishes. Cells were cultured for 4 and 24 h with media containing PDGF BB or PDGF BB + 2 µM WTA to block vimentin polymerization. After incubation, samples were fixed and stained for F-Actin (red), Nuclei (blue). Image width is 465 µm. (**B**) Graphs are data from 3 different experiments. (* *p* < 0.05, ** *p* < 0.01, **** *p* < 0.0001).

**Figure 8:**
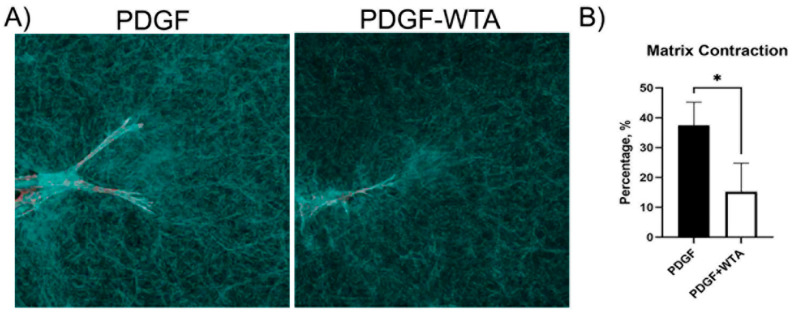
Reduction in traction force and global matrix contraction. (**A**) Confocal reflection microscopy was used to visualize cell–collagen matrix interactions. Cells cultured with WTA showed a reduction in the compaction of matrix, which can be visualized as a reduction in the accumulation of collagen fibers around cell extensions. Image width is 185 µm. (**B**) Global matrix contraction was also reduced when vimentin polymerization was blocked with WTA (* *p* < 0.05), indicating a reduction in tractional force generation. Data are the average of 3 different experiments with 3 different samples per experiment.

Previous studies have demonstrated that disrupting vimentin assembly using Withaferin can modulate cell spreading, migration, and mechanical activity [18,20,21,22,30,31]. In the current study, we found that treatment of corneal fibroblasts with Withaferin (WTA) inhibited vimentin filament assembly and localization to cell extensions (Figure S3). This resulted in significantly reduced cell spreading and polarization in response to PDGF by 24 h after treatment (Figure 7). Withaferin also inhibited cell-induced matrix reorganization in 3D collagen matrices, as indicated both by confocal reflection imaging (Figure 8A) and a collagen matrix contraction assay (Figure 8B)

### 3.5. Vimentin Is Not Requred for Myofibroblast Transformation of Corneal Keratocytes

**Figure 9:**
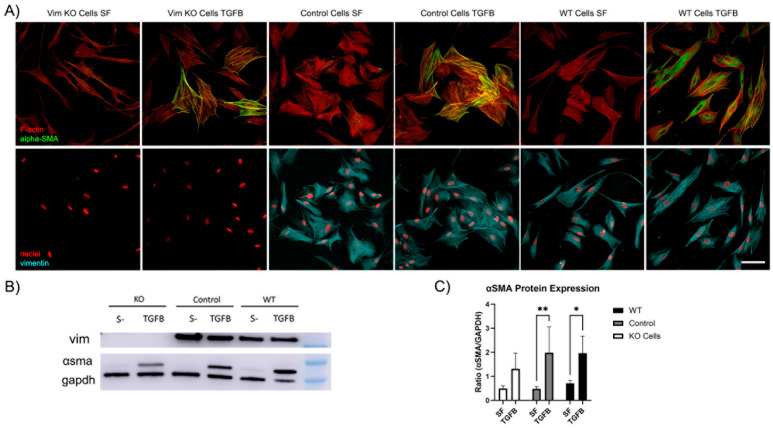
Vim KO cells transform into myofibroblasts. (**A**) Representative pictures of corneal fibroblasts cultured on 2D substrates for 6 days in serum-free media (SF) with or without TGFβ1. After incubation, cells were fixed and labeled for F-actin (red in top panel), αSMA (green in top panel), vimentin (cyan in bottom panel), and nuclei (red in bottom panel). Bar is 100 μm. (**B**) Western blot shows αSMA protein expression following incubation in TGFβ for all cell types. (**C**) Quantification of protein expression from Western blots. Data from 3 independent experiments. (* *p* < 0.05, ** *p* < 0.01).

Recent evidence has shown that VIFs may play a role in the development of corneal fibrosis by inhibiting myofibroblast transformation in a mouse model [20,27]. In order to determine whether vimentin is required for myofibroblast transformation of human corneal fibroblasts, Vim KO and wild-type cells were cultured on collagen-coated dishes in the presence of TGFβ (a promoter of myofibroblasts transformation) and in basal SF media. Myofibroblasts are reparative cells that synthetize, secrete, and reorganize the ECM. Not all myofibroblast are alike; however, they are characterized by the universal expression of αsmooth muscle actin (αSMA) [32]. Interestingly, corneal fibroblasts were able to transform into myofibroblasts in response to TGFβ in the absence of vimentin, as indicated by the development of prominent F-actin stress fibers and positive αSMA labeling (Figure 9A). However, the amount of positive αSMA labeling appeared to be reduced as compared to WT or control cells.

To quantify αSMA protein expression, we conducted Western blot analysis. Western blot images confirmed that αSMA protein was expressed in Vim KO cells (Figure 9B). Quantitative analysis indicated that the amount of αSMA expression induced by TGFβ was reduced in Vim KO cells, consistent with our imaging results.

### 3.6. Proteomics Shows Similar Expression Profiles for KO and Wild-Type Cells

As shown above, Vim KO cells expressed αSMA protein when cultured with TGFβ, albeit at lower levels than controls. Next, we wanted to investigate whether Vim KO cells express other proteins associated with myofibroblast transformation of wild-type cells. To answer this question, we conducted proteomics analysis of Vim KO, control, and wild-type cells following culture in TGFβ. TGFβ is a growth factor known to induce myofibroblast transformation in vivo and is produced by macrophages, epithelial cells, and fibroblasts in many organs and tissues [33]. TGFβ is also known to promote expression of extracellular matrix (ECM) proteins such as collagen 1 and V, fibronectin, and the proteoglycans biglycan and versican, all of which are involved in wound repair and remodeling [33,34,35]. In addition, TGFβ is known to upregulate tropomyosins, proteins which are responsible for incorporating αSMA into stress fibers [36]. TGFβ also increases the induction of connective tissue growth factor, a potent stimulator of myofibroblast differentiation and ECM production [37].

**Figure 10:**
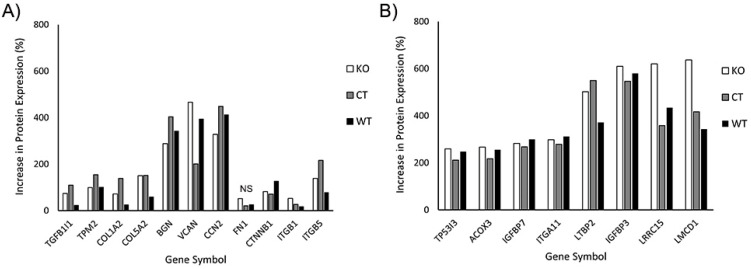
Increase in protein expression induced by TGFβ1 (as compared to SF media). All 3 cell types were incubated for 6 days with media containing TGFβ or SF basal media. Subsequently, protein was extracted and processed for mass spectrometry. (**A**) Increase in expression of known proteins associated with corneal myofibroblasts. (**B**) Additional proteins that were found to have at least twofold increases in all 3 cell types.

In our initial analysis, we evaluated the effects of TGFβ1 on expression of proteins known to be associated with myofibroblast transformation. This included transforming growth factor beta 1-induced transcript 1 (TGFB1I1), which regulates the TGFβ signaling pathway; Tropomyosin 2 (TPM2), collagen 1 and V (COL1A2, COL5A2), fibronectin (FN1), the proteoglycans biglycan and versican (BGN, VCAN), and connective tissue growth factor (CCN2). We also included additional proteins reported to be upregulated in corneal myofibroblasts, such as β-catenin (CTNNB1), and integrin β1 and β5 (ITGB1, ITGB5) [33,38]. Figure 10A shows the percentage increase in the abovementioned proteins induced by culture in TGFβ1. Note that increases in protein expression were observed in WT, control and Vim KO cells, suggesting that Vim KO cells follow a similar differentiation pathway as control and wild-type cells during myofibroblast transformation.

In our proteomics analysis, we found 8 additional proteins that had at least a twofold increase in expression following culture in TGFβ1 (Figure 10B). Except for acyl-CoA oxidase 3 (ACOX3) protein, all other 7 proteins have been reported to play a potential role in myofibroblast behavior and/or fibrosis in other cell types. Specifically, insulin-like growth factor is often found embedded in the ECM where it interacts with matrix proteins, and insulin-like growth factor binding proteins such as IGFBP7 play an essential role in this process [39]. Integrins such as ITGA11 are essential for strong cell–matrix attachments to resist highly contractile forces [40,41]. Tumor protein P53-inducible protein 3 (TP53I3) provides apoptotic resistance to myofibroblasts during fibrosis [42]. LMCD1 has been shown to be highly upregulated by myofibroblasts in order to increase expression of ECM proteins and maintain high levels of αSMA [43]. Latent transforming growth factor binding proteins (LTBP2) are essential for securing TGFβ to the ECM through the latency-associated pro-peptide, LAP [44,45].

Interestingly, LRRC15, a protein previously identified in cancer myofibroblasts [46], was highly expressed in corneal myofibroblasts, both with and without vimentin. LRRC15 associates with various extracellular proteins such as fibronectin and collagen, as well as with integrin β1 (ITGB1) and α11 (ITGA11) [47,48,49]. LRRC15 activates beta1-integrin/FAK signaling to promote ovarian cancer metastasis and is also involved in integrin signaling during fibroblast transformation of periodontal ligament cells [48,50]. However, to our knowledge, it has not been identified previously in corneal fibroblasts or myofibroblasts. Our proteomics data showed that key proteins that may interact with LRRC15, such as collagen, fibronectin, integrin β1, and integrin α11, were also upregulated in response to TGFβ treatment.

In general, the overall pattern of protein expression in response to TGFβ was similar in all three cell types. One protein that is found in fibrotic tissue and facilitates myofibroblast differentiation is periostin, POSTN [38,51]. Interestingly, periostin expression was upregulated only in Vim KO cells, with an increase of 282%; no significant increase was observed in control or wild-type cells.

## 4. Discussion

Vimentin has been associated with myofibroblast transformation of corneal keratocytes and the development of fibrosis in vivo [20,21,27,52], and studies in other cell types suggest that it may regulate several aspects of cell mechanical behavior, including mechanosensing, polarization, and directional migration [13,20,23,53,54,55,56]. However, the role of vimentin can vary depending on cell type, and the effect of vimentin KO has been studied primarily in mouse embryonic fibroblasts. In this study, we explored the role of vimentin intermediate filaments in the spreading of human corneal fibroblasts in 2D and 3D environments, and we also investigated whether vimentin was required for their transition into myofibroblasts. To achieve our goal, we developed vimentin knockout corneal fibroblasts and conducted comparative experiments with wild-type and control cells. Experiments were carried out using a well-established human corneal fibroblast cell line. This cell line has been shown to behave similarly to primary cultures of corneal keratocytes in multiple published studies assessing growth factor responses and mechanical activity [28,57,58,59,60,61].

During cell spreading, there is a complex interactive process between the dynamics of the cytoskeletal filament proteins and the formation of focal adhesion complexes that attach cells to the substrate. Previous studies in other cell types suggest that vimentin null cells retain the ability to spread and exert tractional forces [62] but that vimentin plays a role in regulating cell polarization, as well as the orientation of these forces [54]. In our studies, the loss of vimentin intermediate filaments in corneal fibroblasts did not significantly influence their ability to spread on 2D collagen-coated glass or inside 3D collagen matrices. However, consistent with previous studies, decreased elongation (i.e., polarization) of Vim KO cells was observed in response to PDGF. Our previous studies have shown that corneal fibroblasts develop focal adhesions and apply traction forces to the extracellular matrix to spread and/or migrate [63]. This process is recognized to involve the actomyosin machinery and the dynamics of vimentin in other cell types [64,65]. Our studies using the collagen matrix contraction model show that Vim KO cells and wild-type cells produced a similar amount of matrix reorganization, suggesting that similar amounts of tractional force are generated by the cells. Interestingly, blocking vimentin polymerization using Withaferin had much more significant effects on cell spreading and caused significant inhibition of tractional force generation. Together, these and other data suggest that while vimentin can modulate cell spreading and migration through its phosphorylation and polymerization, compensatory or redundant mechanisms allow for cell spreading in the absence of vimentin. This is also supported by the fact that vimentin KO mice do not show significant developmental abnormalities.

Fibrosis in the cornea produces haze that can be observed at the site of the damaged tissue and impairs corneal transparency [24,25]. Haze is caused in part by transformation of corneal fibroblast to myofibroblasts, which secrete a disorganized, fibrotic ECM [25,26]. Studies with vimentin knockout mice have shown that fibroblast migration, wound contraction, and appearance of myofibroblasts are all delayed during the healing of incisional wounds in mouse embryos [66]. Vimentin has been reported to be overexpressed by myofibroblasts at the site of corneal alkali injury or laser injury in the rabbit [52,67]. The use of Withaferin A (WTA), which blocks polymerization of vimentin, has also been shown to inhibit corneal fibrosis and improve corneal clarity during in vivo wound healing [20]. These and other studies suggest that vimentin could be used as a target to inhibit the development of corneal fibrosis [27,31]. However, one challenge with in vivo studies is that knockout models or WTA treatment can impact multiple cell types in the cornea which are known to interact during healing and may also inhibit corneal fibroblast migration into the wound. Thus, the specific role of vimentin in myofibroblast transformation of corneal keratocytes is difficult to assess in vivo. In our study, absence of vimentin did not completely block myofibroblast transformation, since αSMA protein expression was detected by both Western blot and fluorescent staining in Vim KO cells. Nonetheless, the degree of transformation and amount of αSMA protein was reduced as compared to control cells, consistent with previous studies indicating a role for vimentin in this process. Our results are consistent with previous studies in a mouse vimentin KO model showing that injured corneas also express αSMA; albeit at a lower level and shorter time course than wild-type corneas [21].

In our initial proteomics analysis, we evaluated the effects of TGFβ1 on expression of proteins known to be associated with myofibroblast transformation. This included transforming growth factor beta 1-induced transcript 1 (TGFB1I1), which regulates the TGFβ signaling pathway; Tropomyosin 2 (TPM2), collagen 1 and V (COL1A2, COL5A2), fibronectin (FN1), the proteoglycans biglycan and versican (BGN, VCAN), connective tissue growth factor (CCN2), β-catenin (CTNNB1), and integrin β1 and β5 (ITGB1, ITGB5) [33,38]. Increases in expression of these proteins were observed in WT, control, and Vim KO cells, suggesting that Vim KO cells follow a similar differentiation pathway as control and wild-type cells during myofibroblast transformation.

Our proteomics study also identified eight additional proteins whose expression was highly upregulated in response to TGFβ in both Vim KO and control cells. These include insulin-like growth factor binding proteins 3 and 7 (IGFBP3 and IGFBP7). Insulin-like growth factor (IGF) contributes to fibrosis by stimulating collagen production, and changes in IGF expression have been found in fibrotic tissue. IGF binding proteins (IGFBPs) are deposited in the ECM, and they regulate, at least in part, the biological activity of IGF by mediating access of IGF to its receptor and potentiating its effect in tissues [68]. We found high levels of IGFBP 3 and 7, which have been reported in fibrosis of the lung and liver [69,70]. TGFβ has been shown to increase IGFBP3 production [69,71], which is essential for fibroblast to myofibroblast differentiation [72].

Another protein highly expressed in myofibroblasts was integrin α11. Integrin α11β1 is a collagen receptor and has been reported to be involved in myofibroblast differentiation of other cell types [41,73]. Decreases in the levels of integrin α11 can reduce expression of αSMA, which is essential for the myofibroblast phenotype [73]. The latent transforming growth factor beta binding protein 2, LTBP2, was also highly expressed in both Vim KO and control cells. TGFβ is secreted with a latency-associated pro-peptide, LAP, which in this state is unable to bind its receptors and therefore remains latent. Latent TGFβ-binding proteins bind this complex and secure TGFβ to the ECM [44,45]. LTBP2 is overexpressed in myofibroblasts in fibrotic tissue, and studies have shown that silencing LTBP2 suppresses fibroblast–myofibroblast differentiation, whereas overexpression of LTBP2 promotes pulmonary fibroblast–myofibroblast differentiation even in the absence of TGFβ [74].

Another protein highly expressed in Vim KO and control cells is the tumor protein P53-inducible protein 3 (TP53I3), which is a potent transcription factor responsible for the induction of cell-cycle arrest, apoptosis, and senescence [75]. During pathological wound healing, myofibroblasts have been shown to adopt an apoptotic-resistant phenotype to perpetuate fibrosis [42]. Myofibroblast persistence leads to more ECM deposition, remodeling, tissue contraction, and formation of pathological scars. Increased expression of TP53I3 may help prevent persistent fibrosis through a negative feedback loop.

Recently, myofibroblast populations identified in cancer have been characterized by the expression of the leucine-rich repeat containing protein 15, LRRC15, and are known as LRRC15 myofibroblasts, which share many similarities to corneal myofibroblasts observed during wound healing [76,77]. Interestingly, LRRC15 was highly expressed by both Vim KO and control cells following culture in TGFβ in our study. In cancer, the differentiation of fibroblasts into LRRC15 myofibroblasts is carried out through TGFβ signaling [46]. It is remarkable to note that the cells which transformed into myofibroblasts in our study were not derived from any cancer lineage, suggesting for the first time that LRRC15 is more universal and not just restricted to cancer fibroblast lineage. To determine whether LRRC15 protein expression was specific to HTK cells, bulk RNA-seq data from primary cultures of rabbit corneal keratocytes (NRK cells) were analyzed in a parallel study [78]. The gene-encoding LRRC15 protein was again highly expressed following culture in TGFβ as compared to SF media, indicating that LRRC15 expression is not limited to the corneal fibroblast cell line used in the current study. Additional studies are needed to determine the specific role of LRRC15 in regulating corneal fibrosis and its potential as a therapeutic target.

Another protein that was highly expressed in both Vim KO and control cells was the LIM and cysteine-rich domains 1, LMCD1, which is a transcription factor. Evidence suggests that LMCD1 may facilitate profibrotic gene expression, and in tissue fibrosis, it is found to be consistently elevated in pulmonary and renal fibrosis [43,79]. Depletion of LMCD1 expression has been shown to reduce the expression of ECM proteins and αSMA. On the other hand, high levels of LMCD1 correlate with high levels of ECM and αSMA protein, features of tissue fibrosis [43].

Interestingly, our proteomics analysis also found that periostin, a protein that has been associated with fibrosis and myofibroblast differentiation in other organ systems [38,51], was highly expressed in Vim KO cells but not control cells. The N-terminal region of periostin binds integrins to promote cell adhesion, and the C-terminal region of periostin binds ECM proteins to regulate cell–matrix organization [80,81]. Periostin binding to integrins activates the Akt/PKB- and FAK-mediated signaling pathways. Periostin binding to ECM proteins results in collagen crosslinking and fibrillogenesis, which stiffen the matrix, thereby activating the cells for further extracellular matrix production and regulating the biomechanical properties of connective tissue [82,83,84]. Periostin also plays a role in epithelial-to-mesenchymal transition, EMT [81]. EMT has a critical role not only in tissue and organ differentiation but also in tissue repair, where it can adversely contribute to organ fibrosis through local formation of interstitial myofibroblasts [85,86]. TGFβ, the key regulator for organ fibrosis, is also the best known inducer to trigger EMT in cultured cells [87]. In EMT, typically a switch of intermediate filament usage from cytokeratins to vimentin is observed [86]. It is interesting to speculate that our Vim KO cells are compensating for the lack of vimentin with high periostin protein expression to transform into myofibroblasts. Additional studies are needed to answer this question and to determine the functional role of this protein in corneal myofibroblasts and its relationship to vimentin.

## Data Availability

Data are available upon request.

## Supplementary Materials

The following supporting information can be downloaded at: https://www.mdpi.com/article/10.3390/cells13131094/s1, Figure S1: Vim KO cell spreading in 3D collagen matrices for 4 h in the presence of PDGF and SF media. Figure S2. Western blot showing the effect of double siRNA reverse transfection of vimentin in human corneal fibroblasts. Figure S3. Representative pictures after 4 h of cell spreading of corneal fibroblasts cultured on collagen-coated dishes with and without WTA treatment.
