# Supplementary material for: The Role of Vimentin in Human Corneal Fibroblast Spreading and Myofibroblast Transformation
Source: Cells. 2024 Jun 25;13(13):1094. doi: 10.3390/cells13131094 (PMC11240817; doi:10.3390/cells13131094)
Supplement: Supplementary file 1 [file cells-13-01094-s001.zip › cells-3073353-supplementary.pdf]

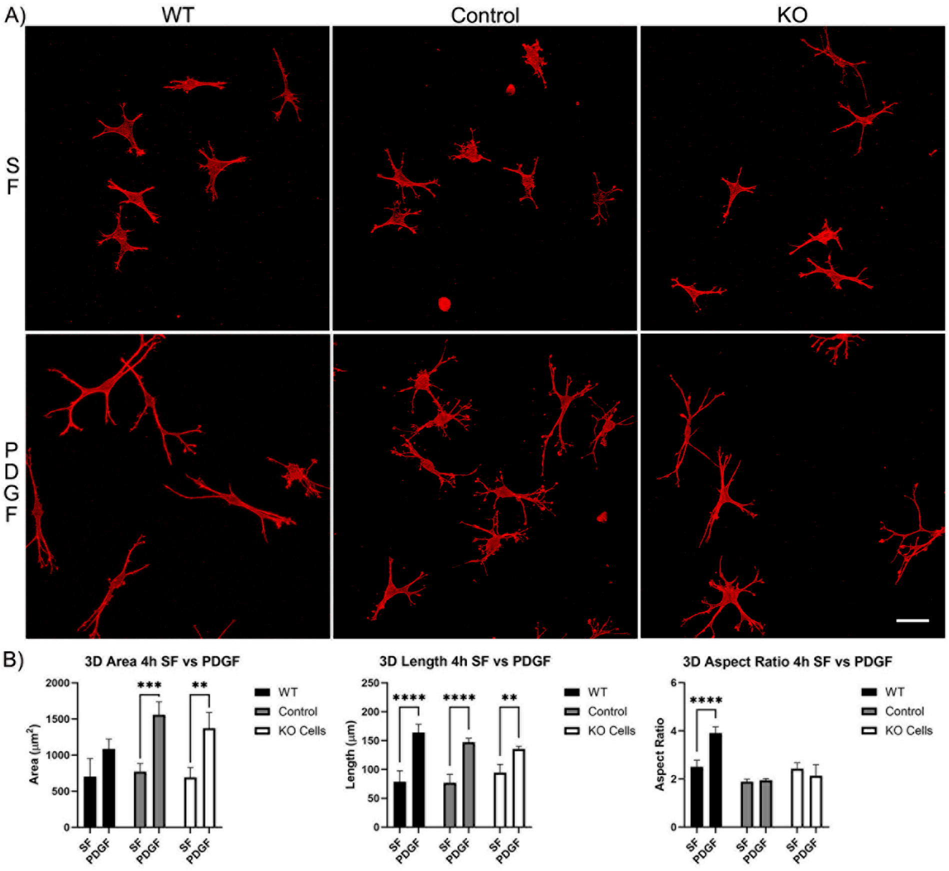

Figure S1. Vim KO cell spreading in 3D collagen matrices for 4 hours in the presence of PDGF and SF media. A) Representative pictures of samples that were fixed and labeled for F-actin. B) Graphs are morphological data from 3 different experiments. Bar is 50 μm.

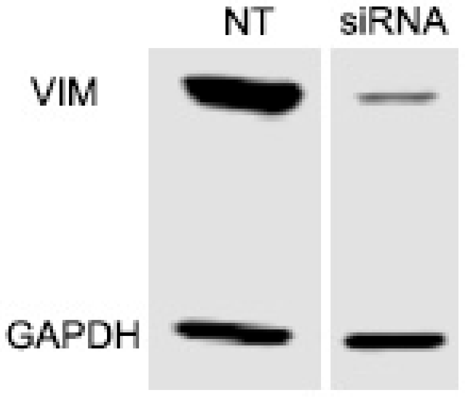

Figure S2. Western blot showing the effect of double siRNA reverse transfection of vimentin in human corneal fibroblasts. Transfection reduced vimentin protein expression by 82.2%.

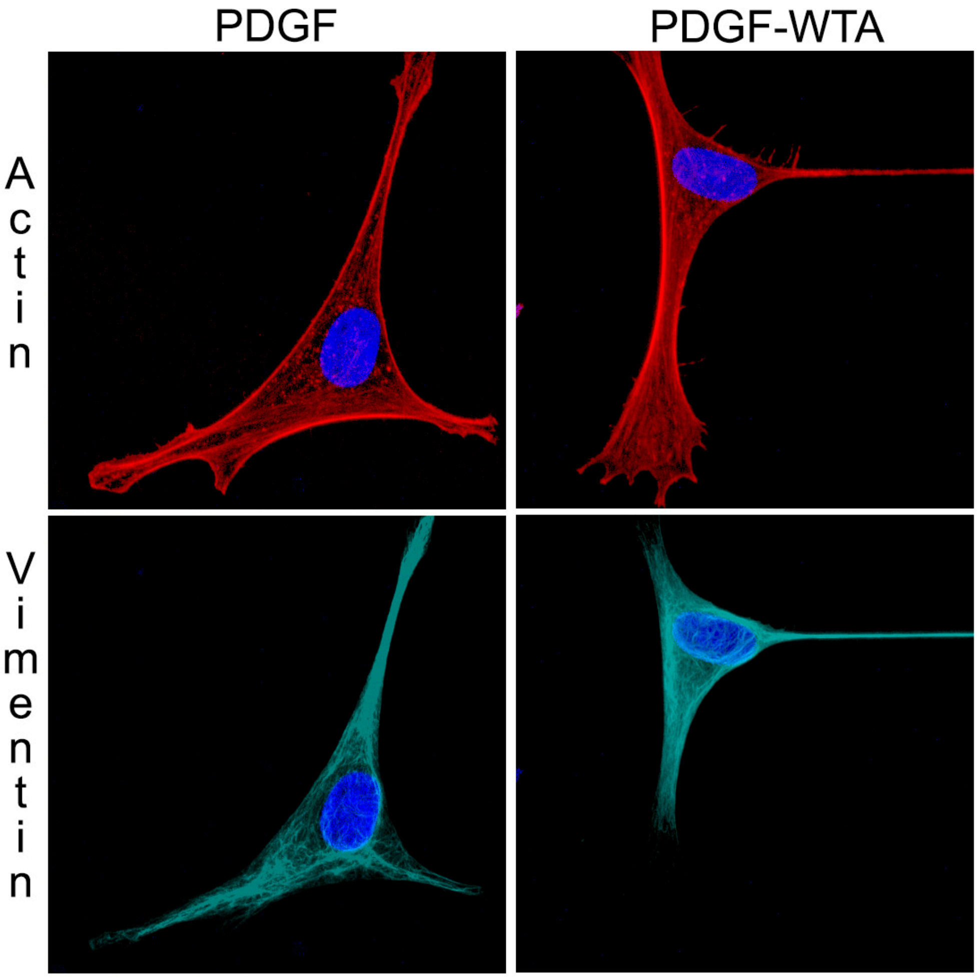

Figure S3. Representative pictures after 4 hours of cell spreading of corneal fibroblasts cultured on collagen coated dishes. Cells were cultured for 4h with media containing PDGF BB or PDGF BB + 2 μM WTA to block vimentin polymerization. After incubation, samples were fixed and stained for F-Actin (red), Nuclei (blue), and Vimentin (green). Cells cultured with WTA showed a retraction of vimentin filaments, which was more concentrated around the nucleus. Image width is 185 μm
